# A multiplicative hazard regression model to assess the risk of disease transmission at hospital during community epidemics

**DOI:** 10.1186/1471-2288-11-53

**Published:** 2011-04-20

**Authors:** Nicolas Voirin, Sylvain Roche, Philippe Vanhems, Marine Giard, Sandra David-Tchouda, Béatrice Barret, René Ecochard

**Affiliations:** 1 Hospices Civils de Lyon, Service d'Hygiène, Epidémiologie et Prévention, Unité Epidémiologie et Biomarqueurs de l'Infection, Lyon, France; 2 Université de Lyon; Université Lyon I; CNRS, UMR5558, Laboratoire de Biométrie et Biologie Evolutive, Equipe Epidémiologie et Santé Publique, Villeurbanne, France; 3 Hospices Civils de Lyon, Service de Biostatistique, Lyon, France; 4 Université de Lyon; Université Lyon I; CNRS, UMR5558, Laboratoire de Biométrie et Biologie Evolutive, Equipe Biostatistiques Santé, Villeurbanne, France; 5 Independent practitioner, Lyon, F-69001, France

## Abstract

**Background:**

During community epidemics, infections may be imported within hospital and transmitted to hospitalized patients. Hospital outbreaks of communicable diseases have been increasingly reported during the last decades and have had significant consequences in terms of patient morbidity, mortality, and associated costs. Quantitative studies are thus needed to estimate the risks of communicable diseases among hospital patients, taking into account the epidemiological process outside, hospital and host-related risk factors of infection and the role of other patients and healthcare workers as sources of infection.

**Methods:**

We propose a multiplicative hazard regression model to analyze the risk of acquiring a communicable disease by patients at hospital. This model derives from epidemiological data on communicable disease epidemics in the community, hospital ward, patient susceptibility to infection, and exposure of patients to infection at hospital. The model estimates the relative effect of each of these factors on a patient's risk of communicable disease.

**Results:**

Using individual data on patients and health care workers in a teaching hospital during the 2004-2005 influenza season in Lyon (France), we show the ability of the model to assess the risk of influenza-like illness among hospitalized patients. The significant effects on the risk of influenza-like illness were those of old age, exposure to infectious patients or health care workers, and a stay in a medical care unit.

**Conclusions:**

The proposed multiplicative hazard regression model could be an interesting epidemiological tool to quantify the risk of communicable disease at hospital during community epidemics and the uncertainty inherent in such quantification. Furthermore, key epidemiological, environmental, host, or exposure factors that influence this risk can be identified.

## Background

Communicable diseases (CDs) such as viral respiratory infections (e.g., influenza virus or rhinovirus infections), viral enteric infections (e.g., hepatitis A virus or rotavirus infections) and bacterial diseases (e.g., group A streptococcal or meningococcal diseases) generally spread throughout the community, from person to person, through hand contact, respiratory route, or fecal-oral route. During community epidemics, these infections may be conveyed to hospital and transmitted to hospitalized patients.

Hospital outbreaks of CDs have been increasingly reported during the last decades and have had significant consequences in terms of patient morbidity, mortality, and associated costs [1,2]. Investigating and analyzing series of hospital outbreaks help identifying the sources of infection, the hosts', and the environmental factors that promote these outbreaks [2,3] and preventing the occurrence of additional cases; besides, these studies increase the knowledge about the disease and improve control measures to avoid future outbreaks.

However, in the hospital setting, the information during an outbreak is mainly descriptive and the measures of disease occurrence remain imprecise. When little data are available, modeling of hospital-acquired infections relies on mathematical models to assess qualitatively the dynamics of CDs and the effects of control measures [4,5]. Conversely, when observational data are existing, it becomes possible to actually quantify the effects. But quantitative studies need to take into account the epidemiological process outside, the hospital and host-related risk factors of infection and the role of other patients and healthcare workers (HCWs) as sources of infection. Because the risk of CD of a patient in a unit of time depends on the number already affected by the CD [6], a distinction between susceptibility and exposure to infection is required to correctly estimate the risk of infection [7]. This may be achieved by incorporating information related to exposure to infection among the explanatory variables. Because exposure to infection changes over time, exposure changes should be treated as time-dependent variables. Estimating parameters and the associated uncertainty is important to answer epidemiological questions that are specific to the hospital setting [4].

We propose here a subject-specific multiplicative hazard regression model to analyze the risk of CD among hospitalized patients. This model includes community CD incidence data and can be used to estimate the relative effects of a stay in a specific hospital ward, host factors and exposure to infection on the risk of CD. Using influenza-like illness (ILI) data collected at hospital during the 2004-2005 influenza season, the analysis shows how to apply the model to assess the risk of ILI among hospitalized patients during community ILI epidemics.

## Methods

### Model rationale

Once a CD has been imported at hospital, it may be of interest to assess if, for a given patient, the risk of CD at hospital differs from the risk of CD if this patient would have stayed at home. For this, we adopted a counterfactual approach and used observed data from the community epidemics to estimate the relative risk of CD at hospital compared to the community. Because the risk of CD for a given patient may depend on the ward he/she stayed, on his/her propensity to acquire the CD and on how he/she is exposed to contagious persons at hospital, the model also includes such variables that are observed at hospital. Then, the model allows 1) estimating the relative risk of CD at hospital compared to the community and 2) studying the hospital-, host- and exposure-related factors that modulate this relative risk.

### Model specifications

The proposed statistical model belongs to the class of multiplicative hazard regression models. The risk of CD at time *t *for a given patient *i *of age *a *with hospital-related, host and exposure characteristics **z **can be modeled as follows:

In this model, λ_C_(*t*_*i*_,*a*_*i*_) represents the expected risk of CD at time *t *for that patient *i *in the community and *G*(*t*_*i*_, **z**_*i*_) corresponds to hospital-, host- and exposure-related effects associated with the elements of covariate vector **z **for that patient *i *at time *t*. The hospital-, host- and exposure-related effects act multiplicatively on the risk of CDs. In the model, *t *denotes actual calendar time with *t *= 0 being the date of start of the study period.

### Expected risk of CD in the community

The expected risk λ_C_(*t*_*i*_,*a*_*i*_) is obtained from relevant general-population diseases statistics using external sources such as surveillance-based age-specific diseases incidence rates. This approach is known as a counterfactual approach in which the expected risk in the community is defined as the risk among the same patients if they have stayed at home. During the period of community epidemics, hospitals are continuously exposed in time to the risk of diseases imported from outside, so that the expected hazard need to be indexed on time. The confounding effect of age is taken into consideration in λ_C_(*t*_*i*_,*a*_*i*_) for the following reasons. First, young children and elderly people are generally supposed to be more susceptible to CDs than young and middle-aged adults because the natural immunity and the resistance to infection are associated with age. Second, young and middle-aged adults are likely to be exposed to infectious children in their households or to other infectious adults with specific risks of CD [8-10]. Third, because vaccination coverage changes with age, different levels of induced immunity to CDs are expected according to age. All the former reasons led us to allow the model controlling for age in the expected risk.

### Hospital, host and exposure effects

We further assume that three hospital-related effects are involved in *G*(*t_i_*, **z**_*i*_): 1) an effect associated with being at hospital, 2) an effect associated with the observed susceptibility of the patient to infection when he/she is at hospital, and 3) an effect associated with his/her observed exposure to infectious persons met at hospital. In the model, these effects are separately considered to allow estimations of their relative contributions to the risk of CD. These three effects at time *t *for a given patient *i *of age *a*, admitted at time *τ *in hospital ward *x*, with susceptibility characteristics *s *and exposure ψ up to time *t *can be modeled as follows:

Expression ν_H_(*t*_*i*_,*x*_*i*_,*τ*_*i*_) represents the effect of being at hospital in ward *x *at time *t *for patient *i *admitted at time *τ*. Expression ν_S_(*t*_*i*_,*s*_*i*_) represents the effect associated with the propensity of patient *i *to acquire a CD according to his/her susceptibility characteristics *s*. Finally, ν_E_(*t*_*i*_,ψ_*i*_) represents the effect of exposure observed at hospital, ψ, of patient *i*. These 3 effects are supposed to act multiplicatively on the risk of CDs. Each of these 3 model's components is detailed below. Written exclusively at the patient level, the following formulas will no more mention index *i*.

### Effect of being at hospital

When a patient is at hospital, several hospital-related effects may change his/her risk of CD. First, he/she is assumed to be isolated and partially protected. Second, this potential protection may depend on the hospital ward (e.g., surgery, intensive care unit, etc.). Third, his/her risk may depend of the day of admission. The interplay of these three effects is modeled with ν_H_. For an given patient admitted at time *τ*, the effect of being at hospital in ward *x *may be modeled as follows:

where β_x _is the effect of being at time *t *in ward *x*, and f(*t*-τ) the effect of the length of stay until time *t *on the risk of CD. For example, f might be a polynomial function.

Parameter β_0 _combines the effect of being at hospital and the effects associated with patient's unmeasured susceptibility and unobserved exposure. All sources of infection (patients, HCWs, or visitors) are usually imperfectly identified; thus, through β_0_, the model allows transmission from unobserved or unobservable sources such as symptomatic infectious persons unnoticed during observation, asymptomatic infectious persons, or asymptomatic carriers. Parameter β_0 _may be negative or positive. A negative value can be interpreted as a decrease in the risk of CD in hospital compared to the community, due for example to isolation, whereas a positive value can be interpreted as an increase in this risk at hospital compared to the community, due for instance to a higher unobserved exposure or a higher susceptibility to infection at hospital.

### Effect associated with observed susceptibility to CDs

Susceptibility characteristics can be added to the model through ν_S_(t, **s**) which represents the propensity of a patient to acquire a CD when he/she is at hospital. For a given patient, susceptibility characteristics measured by covariate vector **s **at time *t *may be included in the model using:

where **β**_S _is the vector of unknown coefficients to be estimated and represents the effects of the elements of covariate vector **s** acting multiplicatively on the risk of CDs. Examples of susceptibility covariates are age at admission, the presence of an immunodeficiency-related disease, or the use of immunosuppressive drugs. Here, age at admission is included in the hospital covariates to capture effects associated with the care of an older patient compared to a younger patient, which may be associated to a different exposure to the risk of disease.

### Effect associated with observed exposure at hospital

When a patient is at hospital, he/she becomes potentially exposed to infectious persons within the institution. The effect of hospital exposure a given patient at time *t *can be modeled by:

where ψ(*t*) represents the observed exposure to infectious persons at hospital.

To define exposures compatible with a transmission, we assume a maximum incubation period of the CD of *J *days. Then, exposure of a patient can be defined as the presence in the same ward of an infectious person during the *J*-day period preceding *t*. During the period of exposure to an infectious patient or HCW, transmission of CD is possible by direct contact, indirect contact, and airborne transmission, whatever the relative importance of each transmission mode. Infected persons remain infectious for a given period and in the following, we assumed a maximum infectious period of *K*-day.

At hospital, patients may be exposed to infectious patients and infectious HCW and for a given patient, exposure observed within hospital at time *t *can be written as follow:

where *P*_*t *_(or *H*_*t*_) take value 1 if the patient is exposed to at least one identified infectious patient (or HCW) during period [*t *- *J*; *t *- 1] and value 0 otherwise. Here, β_P _and β_H _represent the effects of observed exposure at hospital to infectious patients (*P*_*t *_= 1) or infectious HCWs (*H*_*t *_= 1). The model is written with exposure to infectious patients or infectious HCWs, but other sources of infection, such as infectious visitors, can be included in the same way.

### Inference method and estimation

The method of estimation uses a standard manipulation for estimating the parameters of a multiplicative hazard model, by discretization of the time scale and recasting the estimation within the context of Poisson regression [11,12]. It approximates the contribution of each patient to the full log-likelihood by a sum of Poisson terms on time intervals that are sufficiently small for the assumption of a constant rate to be acceptable. Usually, at our hospital, patients are admitted in the afternoon and discharged in the morning. HCWs work either in the morning or the afternoon; thus, a half-day was considered as the width of the time interval. Within a half-day, the effects of exposure to infection and other covariates can be assumed constant. The likelihood can be considered as deriving from a generalized linear model with the outcome "diseased or not" (i.e. 1 or 0), a Poisson distribution function, a log-link function and log(λ_C_(*t*_*i*_,*a*_*i*_)) as an offset. Patients participated to the risk set since the half-day of admission at hospital and follow-up was censored at discharge or at the time of CD onset.

### Data on influenza-like illness used for application

The data originated from 3 sources. The first source is a prospective observational study carried out between November 15, 2004 to April 15, 2007 at Edouard Herriot Hospital in Lyon, France [13]. A total of 36 adult short-stay units participated on a voluntary basis, 12 with 224 beds in 2004-05, 29 with 493 beds in 2005-06, and 30 with 537 beds in 2006-07. During the study, each participating ward was daily followed-up to detect ILI cases; i.e., patients or HCWs presenting with fever (≥ 37.8°C) and cough or sore throat. A non-case was defined as a patient or a HCW free from ILI during the study period. For each case, demographic, medical, and hospitalization data as well as clinical observations related to ILI were recorded through a questionnaire administered by a member of the infection control team. However, only the date of ILI onset for each case was considered here. Patients presenting with ILI at admission were excluded from the analysis, assuming they were not at risk of hospital-acquired ILI. Patients participated to the risk set since the half-day of admission at hospital and follow-up was censored at discharge or at the time of ILI onset (i.e. assuming patients were no more at risk of ILI). A patient re-admitted at hospital participated again to the risk set except if he or she presented ILI during a previous stay or at the time of re-admission. For the present analysis, only data from the season 2004-05 (12 wards) were used. The hospital institutional review board approved the study. All ILI cases (patients and HCWs) received printed information and signed an informed consent form.

As second source, all hospital data (admission and discharge dates, place and immunodeficiency-related diagnoses) of cases and non-cases were extracted from the hospital's information system. Data on periods of work for HCWs were included.

The third source comprised community data on the number of ILI incident cases available from the national surveillance network [14]. These are estimations of the weekly numbers of new ILI cases in the Rhône-Alpes population grouped by 5-year age categories. The data were completed with regional population figures by 5-year age strata obtained from the INSEE (Institut National de la Statistique et des Études Économiques).

We calculated weekly ILI incidence rate in the Rhône-Alpes region by age group by dividing the weekly number of new ILI cases in the Rhône-Alpes region by the Rhone-Alpes population figures. Incidence rates of ILI from the community population were then applied week by week to the age structure of the patients' population (i.e. using indirect standardisation) to calculate λ_C _(*t,a*).

Over a total of 56,826 half-days (4,059 weeks) of follow-up, 24 incident cases of ILI were observed among 4,484 patients at risk of ILI. The incidence rate of ILI at hospital was 5.91 (95% confidence interval (95% CI) 3.96-8.82) per 1,000 patient-weeks.

### Model applications

In this section and based on the data presented in the previous section, we present how the model can be used to assess the risk of ILI among hospitalized patients during community ILI epidemics. The following analysis was performed:

1. *Effect of being at hospital*. To investigate whether the risk of ILI in hospitalized patients was higher than their expected risk in the community, the following model form was used:

with ν_H _= exp(β_0_) representing, for a given patient at time *t*, the hazard ratio (HR) of acquiring ILI at hospital vs. in the community.

To further studied whether the specialty of the wards might affect the risk of ILI, the model can be modified as follows:

with ν_H_(*t,x*_*t*_) = exp(β_0 _+ β_x_*x*_*t*_) and *x*_*t *_being a variable with 4 categories; one for each specialty of the ward (i.e. surgery, medical care, gynecology and intensive care) where a given patient was at time *t*.

2. *Effect associated with observed susceptibility to ILI*. To examine how sex, age at admission, and impaired immune status might modify the risk of ILI, the model was written as follows:

with ν_H _= exp(β_0_) and, for a given patient at time *t*, ν_S_(*t*, **s**) = exp(**β**^'^_S_**s**_*t*_) where **s **is the vector of covariates representing sex (male, female), age at admission (18-34, 35-64, and ≥ 65 years old) and impaired immune status (yes, no).

3. *Effect associated with observed exposure at hospital*. To investigate how exposure to infectious patients and/or HCWs may affect the patients' risk of ILI, we assumed a maximum incubation period of ILI of *J *= 5 days [15] and a maximum infectious period of ILI of *K *= 6 days starting one day before symptom onset [15]. We used the following model:

with ν_H _= exp(β_0_) and, for a given patient at time *t*, ν_E_(*t*,ψ) = exp (ψ(*t*)) with ψ(*t*) = β_P_*P*_*t *_+ β_H_*H*_*t *_+ β_PH_*PH*_*t*_. The variables *P*_*t *_and *H*_*t *_represent the presence of at least one infectious patient or one infectious HCW, respectively, in the 5 days preceding *t*. The variable *PH*_*t *_represented the simultaneous presence of at least one infectious patient, *P*_*t*_, and at least one infectious HCW, *H*_*t*_, in the 5 days preceding *t*.

We present estimations for the 4 separate models. Modelling was done using a strategy of selecting variables based on epidemiological knowledge and no actual model selection based on significance was done. For our application, November 15, 2004 (week 47) was the starting time (*t *= 0).

## Results

### Effect of being at hospital

**Figure 1:**
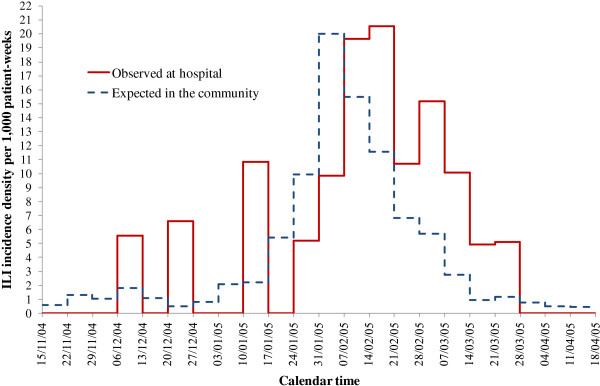
**Observed risk of ILI λ(*t*) and expected risk of ILI λ_C_(*t,a*) during influenza season 2004-2005**.

The weekly incidence rates noted at hospital λ(*t*) and expected in the community λ_C _(*t*,*a*) are shown in Figure 1. The graph suggested comparing the areas under the 2 curves, providing a way to investigate whether being at hospital modulated the patients' expected risk of ILI. During the study period, 24 cases were observed at hospital and 18.07 cases were expected among the 4,484 patients if they have stayed at home.

The HR of acquiring ILI at hospital vs. in the community was ν_H _= 1.33 (95% CI: 0.89-1.98, p = 0.16) which suggested, despite the lack of statistical significance, that during the study period the risk of acquiring ILI would be higher at hospital than in the community.

We further studied whether the specialty of the wards might affect the risk of ILI. Twelve hospital wards, distributed in 4 specialties, participated in the study (Table 1).

**Table 1:** Numbers of ILI cases observed at hospital and expected in the community according to the ward and its medical specialty.

Ward	Specialty	Number of observed ILI cases at hospital		Number of ILI cases expected in the community		Number of half-days of hospital stay	
		By ward	By specialty	By ward	By specialty	By ward	By specialty
W169	Surgery	1	1	1.43	2.96	4,587	9,320
W227	Surgery	0		1.53		4,733	
W109	Medical care	1	22	0.78	6.82	2,541	27,104
W113	Medical care	1		0.78		2,586	
W117	Medical care	1		1.80		5,842	
W230	Medical care	1		1.55		5,675	
W245	Medical care	6		1.15		5,006	
W292	Medical care	12		0.76		5,454	
W235	Gynecology	0	1	3.71	7.15	8,376	16,289
W236	Gynecology	0		2.11		4,918	
W237	Gynecology	1		1.33		2,995	
W134	Intensive care	0	0	1.15	1.15	4,113	4,113

Heterogeneity of the risk of ILI between specialties was assessed by comparing the deviances of the models with vs. without ν_H_(*t,x*_*t*_). The deviance was 31.83 for 3 degrees of freedom (p < 0.01), indicating that the risk of ILI differed among specialties.

Compared to medical wards, the HR of ILI was 0.10 (95% CI 0.01-0.78, p = 0.03) for surgery and 0.04 (95% CI 0.01-0.32, p < 0.01) for gynecology; i.e., a significantly lower risk of ILI was observed in surgery and gynecology units compared to medical units.

### Effect associated with observed susceptibility to ILI

Being a female did not significantly affect the adjusted HR of ILI (2.03, 95% CI: 0.82-5.02, p = 0.13). Compared to that of the 18-34 years age category, the HR for ages 35-64 and ≥ 65 years were 4.04 (95% CI: 0.78-20.82, p = 0.10) and 14.35 (95% CI: 3.09-66.69, p < 0.01), respectively, indicating an increase in the risk of ILI with age at admission. Compared to less susceptible patients, those with impaired immunity had a HR of ILI of 1.47 (95% CI: 0.63-1.44, p = 0.37).

### Effect associated with observed exposure at hospital

The results are reported in Table 2 and suggest that being exposed to infectious patients, infectious HCWs or both significantly increases the risk of ILI compared to the absence of proven source of infection at hospital.

**Table 2:** Numbers of ILI cases observed at hospital and expected in the community according to level of exposure to infectious patients or HCWs observed at hospital.

Exposure	Number of observed ILI cases at hospital	Number of ILI cases expected in the community	Hazard ratio of ILI (95% CI)	p value
No infectious patient or HCW documented	12	16.01	1.00	-
At least 1 infectious HCW but no infectious patient	1	0.30	4.44 (0.58-34.18)	0.15
At least 1 infectious patient but no infectious HCW	7	1.26	7.42 (2.92-28.84)	< 0.01
At least 1 infectious HCW and 1 infectious patient	4	0.50	10.63 (3.43-32.95)	< 0.01

## Discussion

The multiplicative hazard regression model developed here allows assessing the risk of CDs among hospitalized patients. This model can be used to quantify the risk of CD at hospital according to three axes: i) explore whether the risk of CD at hospital differs from the one in the community and whether there is heterogeneity among wards; ii) identify host factors facilitating CDs; iii) identify the main routes of CD transmission at hospital.

The application of that model to ILI integrates simultaneously information on community ILI epidemics, hospital wards, susceptibility to the disease, and exposures occurring at hospital. In this application, it was shown that being hospitalized might increase the patient's risk of ILI compared to the community, that significantly higher risks were observed in medical wards than in other specialty wards, and that infectious patients may represent an important route of transmission to other patients.

Identifying at risk hospital areas, high risk groups of patients, and primary interventions according to the route of transmission may be particularly helpful in managing and efficiently controlling person-to-person spread of CDs at hospital. Some wards may present high risks of CDs while others might be rather protective. Identifying the latter is important to isolate specific patients such as the immunosuppressed ones. In fact, the functioning of a ward (e.g., specialty, infection control measures, vaccination coverage of patients and HCWs, distance between beds, availability of hand-washing materials for staff, etc.) rather than its location is probably of interest. Identifying and comparing the routes of transmission could help define and prioritize control measures such as closing the ward, restricting the visits, avoiding contact between susceptible patients and infectious persons, or isolating infectious persons (patients or HCWs) to protect patients, especially the more susceptible ones.

Some statistical approaches have been proposed to model infectious diseases data in a regression framework [16-18] providing often a simple method making use of existing software. In addition, some hospital-, ward- and subject-specific approaches have been proposed [17,19-23] to analyze hospital infection data. However, compared to these approaches, the model presented here is, to our knowledge, among the first to include simultaneously the specificities of the hospital setting, namely ward heterogeneity, host-factor susceptibility and separated patients and HCWs exposures. This allows estimating separately these effects. More specifically, the epidemiological process outside, which may influence the epidemiological process inside, is explicitly taken into account in our model using known data on the risk due to a circulation of the disease in the community.

Due to the high fluctuations in the number of patients (admission and discharge), of HCWs work hours and of HCWs and patients exposure, subject-specific models may be preferred for hospital outbreak modeling. In our model, exposure was defined, at the patient level and for each day of hospitalization, as the presence of an infectious person in the same ward during the days preceding time *t*. Within the context of CDs it seems more appropriate to consider exposure rather than contact because it is not easy to determine which contact among many led to the infection. This definition includes all types of disease transmission and may be refined according to the studied pathogen.

However, the model assumptions may limit its scope. If each person included in the study could be individually followed-up, it would be possible to know who contacted whom, where, when, and for how much time. The model assumes that the number of contacts during exposure was sufficient to induce infection, which may not be the case because the contacts were not observed. Each hospital outbreak is unique, with specific events leading to the epidemic. Thus, the results of a single epidemic should not be generalized to other contexts. However, the model could be a valuable tool for evaluating and comparing outbreaks between years in the same area. Besides, there was a discrepancy between the model presented in its most general form and the data that may not have been rich enough to demonstrate simultaneously all its features. The low number of incident events (only 24 ILIs) did not allow estimating all the parameters linked with hospital-related variables, susceptibility, and exposure. It could be interesting to apply the model to a larger dataset.

## Conclusions

The proposed multiplicative hazard regression model could be an interesting epidemiological tool to identify key epidemiological, environmental, host, or exposure factors that increase or decrease the risk of CD in other confined settings such as kindergartens, schools, workplaces or day-care facilities where individuals and groups interact closely. In the hospital setting, this model could be a valuable tool to assess the risk of CD among patients in order to improve everyday infection control and management of local outbreaks.

## Competing interests

NV and PV have received PhD and research grant contributions respectively, from sanofi pasteur. All other authors have no conflict of interest to declare. The funders had no role in study design, data collection and analysis, decision to publish, or preparation of the manuscript.

## Authors' contributions

NV, SR, PV, MG, SDT and RE designed the model. PV and BB provided access to the data. All authors participated to the interpretation of data. All authors have been involved in drafting the manuscript and in revising it critically for important intellectual content. All authors have given final approval of the version to be published.

## Pre-publication history

The pre-publication history for this paper can be accessed here:

http://www.biomedcentral.com/1471-2288/11/53/prepub
